# Vitamin C Alleviates the Negative Effects of Heat Stress on Reproductive Processes by Regulating Amino Acid Metabolism in Granulosa Cells

**DOI:** 10.3390/antiox13060653

**Published:** 2024-05-27

**Authors:** Abdul Sammad, Tanveer Ahmed, Khair Ullah, Lirong Hu, Hanpeng Luo, Piniel Alphayo Kambey, Shah Faisal, Huabin Zhu, Yinxiong Li, Yachun Wang

**Affiliations:** 1 State Key Laboratory of Animal Biotech Breeding, National Engineering Laboratory for Animal Breeding, Key Laboratory of Animal Genetics, Breeding and Reproduction of Ministry of Agriculture and Rural Affairs, College of Animal Science and Technology, China Agricultural University, Beijing 100193, China; abdul@gibh.ac.cn (A.S.); b20193040324@cau.edu.cn (L.H.); luohanpeng@cau.edu.cn (H.L.); 2 Center for Health Research and Guangdong Provincial Key Laboratory of Biocomputing, Guangzhou Institutes of Biomedicine and Health, Chinese Academy of Sciences, Guangzhou 510530, China; tanveer@gibh.ac.cn (T.A.); khair@gibh.ac.cn (K.U.); kambey@gibh.ac.cn (P.A.K.); shahfaisal11495@gmail.com (S.F.); 3 University of Chinese Academy of Sciences, Beijing 100049, China; 4 Institute of Animal Science, Chinese Academy of Agricultural Sciences, Beijing 100193, China; 5 Guangdong Provincial Key Laboratory of Stem Cell and Regenerative Medicine, Guangzhou 510530, China; 6 State Key Laboratory of Respiratory Disease and China-New Zealand Joint Laboratory on Biomedicine and Health, Guangzhou 510530, China; 7 CAS Key Laboratory of Regenerative Biology, Guangzhou Institutes of Biomedicine and Health, Chinese Academy of Sciences, Guangzhou 510530, China

**Keywords:** vitamin C, heat stress, metabolomics, amino acid, catabolism

## Abstract

Heat stress-induced biochemical alterations in ovarian follicles compromise the function of granulosa cells (GCs) and the developmental competence of oocytes. Summer heat stress can have a far-reaching negative impact on overall fertility and reproductive success. Together with the heat stress, the rise of assisted reproductive technologies (ART), potential confounding hazards of in vitro handling and the absence of systemic body support in ART makes it imperative to study the heat stress ameliorative effects of vitamin C under in vitro conditions. Using in vitro heat stress treatment of 43 °C for two hours in bovine GCs, we studied the effects of vitamin C on cell growth, oxidative stress, apoptosis and cell cycle progression together with a comprehensive metabolomics profiling. This study investigates the molecular milieu underlying the vitamin C (VC)-led alleviation of heat-related disruptions to metabolic processes in bovine GCs. The supplementation of VC ameliorated the detrimental effects of heat stress by reducing oxidative stress and apoptosis while restoring cell proliferation. Normal cell function restoration in treated GCs was demonstrated through the finding of significantly high levels of progesterone. We observed a shift in the metabolome from biosynthesis to catabolism, mostly dominated by the metabolism of amino acids (decreased tryptophan, methionine and tyrosine) and the active TCA cycle through increased Succinic acid. The Glutathione and tryptophan metabolism were important in ameliorating the inflammation and metabolism nexus under heat stress. Two significant enzymes were identified, namely tryptophan 2,3-dioxygenase (TDO2) and mitochondrial phenylalanyl-tRNA synthetase (FARS2). Furthermore, our findings provide insight into the significance of B-complex vitamins in the context of heat stress during VC supplementation. This study underscores the importance of VC supplementation in heat stress and designates multiple metabolic intervention faucets in the context of ameliorating heat stress and enhancing reproductive efficiency.

## 1. Introduction

Usually, all living organisms experience heat stress at an ambient temperature exceeding 25 °C. Higher temperatures in the range of 35–40 °C cause significant physiological and biochemical changes in the body [1]. With ever-increasing events of heat waves, even in temperate climatic zones, heat stress is an increasing public health concern. Heat stress particularly poses a serious threat to reproductive function in animals and humans, male and female alike [2]. Moreover, the female reproductive system and ovaries, in particular, make them more susceptible to the adverse effects of increased core body temperature due to heat stress because of their anatomical location [3]. As ovarian follicular dynamics and spermatogenesis are lengthy processes, the carry-over effects of heat stress at an early age or in hot months even extend to gamete quality at a later age and in cold months, respectively [4,5].

The human genome sequence is more similar to that of cattle compared with rodents [6]. Farm animals are important biomedical models, and thus, livestock animals are valuable models for studying infertility in women [7,8]. Most importantly, due to the reasons of ethics, welfare and feasibility, studies using farm animals and derived biomaterials seem valuable in the context of heat stress and infertility. It is noteworthy that, unlike other monogastric livestock species, ruminants can synthesize vitamin C on their own, and 15 to 20 μM of its plasma concentration is reported in cattle [9]. Vitamin C is rapidly declined in disease and stress and, thus, causes low circulatory ascorbic acid [10]. In these states, the body utilizes more vitamin C and, thus, causes oxidative stress and inflammation-mediated damage [11]. In a recent study, vitamin C supplementation in aging female mice models had a positive effect on the ovarian follicle reserves [12]. Alongside other modern management technologies, vitrification, ovum pickup (OPU), in vitro maturation (IVM) and in vitro fertilization (IVF) are becoming increasingly popular for tackling infertility issues in women [13]. Both mural and cumulus GCs have an integral role in reproduction through oocyte nurturing, where cumulus GCs remain attached to oocytes in IVM, and this co-culture is beneficial for the success of reproductive biotechnologies [14,15].

In the absence of a systematic vitamin C supply of circulation and the events of ex vivo handling and culturing conditions, the investigation of vitamin C ameliorative properties and underlying mechanisms using GCs is imperative and will present an insightful simulation in the context of reproductive biotechnologies. It has been well known that vitamin C supplementation augments antioxidant response by upregulating enzymatic and non-enzymatic antioxidants and ameliorating inflammation. Therefore, it will be interesting to investigate the effects of vitamin C on the physical and functional attributes of GCs under in vitro heat stress conditions. Furthermore, the investigation of the unfolding of the metabolic landscape of vitamin C supplementation at the nexus of metabolome under the conditions of acute heat stress will further augment our results and help in the prediction of potential metabolic interventions in the context of heat stress and reproductive biotechnologies.

## 2. Methods

### 2.1. Granulosa Cells Culture and Treatment

Granulosa cells were collected, cultured and treated according to the detailed methods explained in our previous studies [16]. Briefly, GCs were collected from healthy ovaries sourced from Simmental cattle and cultured in media containing DMEM/F12 medium (Thermo Fisher Scientific, Waltham, MA, USA), 10% fetal bovine serum (FBS) (Thermo Fisher Scientific, Waltham, MA, USA) and 1% penicillin–streptomycin under 38 °C and 5% CO_2_ in humidified incubator. Culture medium was changed every 24 h; GCs were fasted with 2% FBS containing media for 12 h. before treatment. GCs were cultured in a fresh, original culture medium (with or without vitamin C) for an additional 6 h at 38 °C and afterward subjected to 2 h of acute heat stress (43 °C). At the same time, control group remained at 38 °C. Cell viability for both groups was measured at 24 h post-heat stress treatment. Cells (1 × 10^6^ in each well) were cultured with different dilutions (50, 100, 200, 300 and 500 μM) of vitamin C (HY-B0166, MedChemExpress, Monmouth Junction, NJ, USA) in 48-well plates, and optical density (OD) value was measured using Cell Counting Kit-8 (Cat. no. CK04, Dojindo Laboratories, Kumamoto, Japan) according to the manufacturer instructions and using an Infinite M200 PRO (Tecan Deutschland GmbH, Crailsheim, BW, Germany) plate reader at 450 nm wavelength.

### 2.2. Cell Growth Assay

Similarly, GCs (1 × 10^4^ in each well) for control, heat stress and heat stress plus vitamin C groups were cultured in 96-well cell culture plates containing DMEM/F12 medium (Thermo Fisher Scientific, Waltham, MA, USA) with 10% FBS (Thermo Fisher Scientific, Waltham, MA, USA) and 1% penicillin–streptomycin under 38 °C and 5% CO_2_ in humidified incubator. Culture medium was changed every 24 h; GCs were fasted with 2% FBS-containing media for 12 h before treatment. GCs were cultured in a fresh, original culture medium (with or without 250 μM vitamin C) for an additional 6 h at 38 °C and afterward subjected to 2 h of acute heat stress (43 °C). At the same time, control group remained at 38 °C. Starting from 0 h, measured using Cell Counting Kit-8 (Cat. no. CK04, Dojindo Laboratories, Kumamoto, Japan) according to the manufacturer instructions and using an Infinite M200 PRO (Tecan Deutschland GmbH, Crailsheim, BW, Germany) plate reader at 450 nm wavelength.

### 2.3. Reactive Oxygen Species

GCs were cultured in culture media containing 250 μM vitamin C in 12-well clear bottom plates (2 × 10^4^ in each well), processed after an hour of post-heat stress recovery at 38 °C and 5% CO_2_ and florescence OD recorded. Heat stress and heat stress plus vitamin C groups were measured using a 6-carboxy-2′, 7′-dichloro-dihydro-fluorescein diacetate staining kit (DCFDA kit (ab113158), Abcam, Cambridge, MA, USA) according to the manufacturer instructions. The absorbance was measured by an Infinite M200 PRO (Tecan Deutschland GmbH, Crailsheim, BW, Germany) plate reader at excitation/emission of 485/535 nm wavelength. 

By employing similar conditions, mitochondrial superoxide-based oxidative stress was measured through MitoSOX^TM^ Red staining kit (cat. no. M36008, Molecular Probes Inc., Eugene, OR, United States, Invitrogen Technologies, Carlsbad, CA, USA) by using Infinite M200 PRO (Tecan Deutschland GmbH, Crailsheim, BW, Germany) plate reader at excitation/emission of 510/580 nm wavelength.

### 2.4. Apoptosis Measurement

GCs were cultured in culture media containing 250 μM vitamin C in 12-well clear bottom plates (2 × 10^4^ in each well), while the control and heat stress alone group had no added vitamin C. After six hrs. of post-heat stress recovery at 38 °C in humidified CO_2_ incubator, the qualitative apoptosis rate of GCs from all three groups was assessed using Annexin V-FITC and PI (propidium iodide) staining kit (Cat. no. G003-1-2, Nanjing Jiancheng Bio Inst., Nanjing, China) according to the kit’s manufacturer protocol. Flow cytometry was performed by FACS Calibur (BD Biosciences, San Jose, CA, USA), and data were analyzed by FlowJo™ v10.4 software. Three replicate vials of both groups were analyzed for apoptosis measurements.

### 2.5. Hormone Measurements

Cells from control, heat stress and heat stress plus vitamin C groups were shifted to 38 °C incubator for six hrs. (recovery at 38 °C in a humidified CO_2_ incubator) after the conclusion of heat stress treatment. After, the culture media from all groups were collected and tested through ELISA kits (Cusabio Technology LLC, Wuhan, China) according to manufacturer protocols for progesterone (Cat. no. CSB-E08172b) and estrogen hormones (Cat. no. CSB-E07280m) concentrations. The absorbance was measured by an Infinite M200 PRO (Tecan Deutschland GmbH, Crailsheim, BW, Germany) plate reader at 450 nm wavelength. 

### 2.6. Cell Cycle Analysis

Cells (2 × 10^4^ in each well) in 12-well clear bottom plates from control, heat stress and heat stress plus vitamin C groups were shifted to 38 °C incubator for six h (recovery at 38 °C in humidified CO_2_ incubator) after the conclusion of heat stress treatment. The cell cycle analysis kit (Cat. no. C1052, Beyotime, Shanghai, China) was used to analyze cell cycle according to the manufacturer’s protocol given in the kit and the method detailed in our previous study [17]. Briefly, cells were fixed in 70% prechilled alcohol overnight, washed with prechilled PBS, PI stained for 30 min at 30 °C and analyzed through BD LSRFortessa™ (BD Biosciences, San Jose, CA, USA). The data obtained from the PE-A channel were processed through FlowJo™ v10.8 software for cell cycle analyses.

### 2.7. Western Blot

GCs were cultured in culture media with or without vitamin C in six-well clear bottom plates (2 × 10^4^ in each well) and treated with heat stress (43 °C) while control group remained at 38 °C in humidified CO_2_ incubator. After six hours of recovery at 38 °C in humidified CO_2_ incubator, cells were collected and processed for protein extraction and Western blotting according to our previous detailed protocol [17]. Briefly, equal amounts of protein transferred to polyvinylidene difluoride membranes, blocked for 60 min and incubated with primary antibodies: anti-Caspase-3 (1:1000, Cat. no. 14220T, Cell Signaling Technology, Beverly, MA, USA), PCNA (1:1000, Cat. No. 13-3900, Invitrogen Technologies Inc.) and β-actin (1:1000, Cat. no. sc47778, Santa Cruz Biotechnology Inc., Dallas, TX, USA) at 4 °C overnight. Subsequently, the membranes were washed and incubated with HRP-conjugated goat anti-mouse IgG Alexa Flour 555 (Cat. no. ab150114, Abcam) and anti-rabbit IgG Alexa Flour Plus 647 (Cat. no. A32733, Thermo Fisher Scientific) secondary antibodies. The enhanced chemi-luminescence detection reagent (Thermo Fisher Scientific Inc., Waltham, MA, USA) was used to visualize the bands. The bands were measured using the Image J version 1.44p software [18], and β-actin was used as a reference protein for normalization.

### 2.8. Statistical Analysis

The data from at least six replicates each for cell proliferation and ELISA and at least three replicates for ROS, apoptosis and cell cycle analysis measurements were utilized in statistical analysis. Visualization of the data and statistical differences among control and heat stress, and heat stress and heat stress plus vitamin C groups were carried out using Graphpad prism 9.0.0 version software. Analysis of variance was performed, and means were compared using Tukey’s honestly significant difference (HSD) test at a 5% level of significance (α = 0.05). All the data represented in the figures are expressed as mean ± S.D.

### 2.9. LC-MS/MS Analysis

Sample preparation for LC-MS/MS, analysis and pre-processing of peaks and metabolome analysis were performed according to our previous detailed methods [19]. In brief, GCs in control (43 °C for 2 h) and treatment (supplemented with 250 μM vitamin C for 43 °C for 2 h) groups in six-well culture plates were allowed to recover for 6 h at 38 °C in a humidified CO2 incubator. Culture medium was quickly collected, and metabolite extraction was performed. Sample replicates and quality controls were run through LC-MS/MS using HSS T3 100 × 2.1 mm 1.8 μm column (Waters, Waltham, MA, USA) on Ultimate 3000 (Thermo Fisher Scientific, Waltham, MA, USA) followed by the analysis employing Q Exactive MS system (Thermo Fisher Scientific, Waltham, MA, USA). For metabolites in both positive and negative ion modes, peak identification, retention time correction and peak area integration were performed through MS-DIAL (version no. 4.9.221218) software [20].

### 2.10. Metabolome Analysis

The MetaboAnalyst 5.0 package [21] was employed to carry out the principal component analysis (PCA) for investigating the clustering trends and outliers for both ion modes metabolites. PCA and partial least square discriminate analysis (PLS-DA) were separately performed for negative and positive modes peak intensities input files. PLS-DA was performed to check the fold change analysis, Student’s *t*-test-based significance, variable important in projection (VIP) and other statistical tests to identify differential metabolites. Furthermore, metabolites with VIP values equal to or greater than 1 were considered as differentially expressed metabolites (DEMs) between respective comparisons. MetaboAnalyst 5.0 online software’s modules of pathway and enrichment analysis were used to determine the biological processes of differential metabolites involvement, where all DEMs from both comparisons were run through small molecule pathway database (SMPDB) version 2.0 [22] and Kyoto Encyclopedia of Genes and Genomes (KEGG) database [23] pathways enrichment analyses. Metabolite–metabolite interaction network sub-module with default settings was used for the interaction analysis among metabolites by using metabolites data input, while these chemical–chemical associations for the metabolites network were extracted from Search Tool for Interacting Chemicals (STITCH) version 5.0 [24]. Additionally, all metabolites from both comparisons involving vitamin C were subjected to MetaBridge 1.7.52 software [25] to obtain the lists of interacting enzyme proteins and corresponding metabolites. After this, we accessed NetworkAnalyst 3.0 software [26] to visualize these enzyme protein networks through generic protein–protein interaction (PPI) network based on Rolland Interactome [27]. The corresponding metabolites were visualized by a separate subjection to metabolite—disease interaction network analysis in network analysis module of MetaboAnalyst 5.0, which is based on human metabolome database (HMDB) version 5.0 [28].

## 3. Results

### 3.1. Physical Parameters of GCs

**Figure 1:**
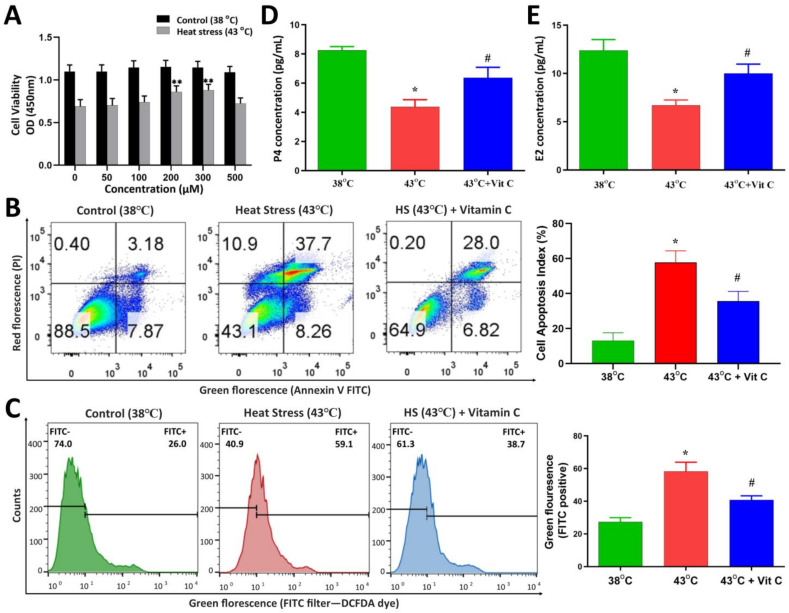
Physical parameters of bovine granulosa cells. Comparison of physical parameters of bovine granulosa cells (GCs) exposed to heat stress (43 °C for 2 h) and vitamin C (Vit C) treatment versus control (38 °C). (**A**) CCK-8-based GCs viability with mean optical densities (OD) measured at 450 nm wavelength are plotted against different concentrations of vitamin C for control and heat stress group, while panels with double asterisks (**) are significantly different (*p*-value < 0.05) from zero micromolar concentration measurement at 0 h. (**B**) Representative flow cytometry plots of control group, heat stress and heat stress plus vitamin C groups. Means comparisons of apoptotic ratios are shown in bar graphs for all three groups by summing up right bottom, right and left upper quadrants of flow cytometry plots for each group. (**C**) Representative green fluorescence of GCs stained with 2′,7′-dichlorofluorescine diacetate (DCFDA) measured via flow cytometry. Relative fluorescence values are shown on the y-axis, and treatment groups are indicated on the *x*-axis. (**D**,**E**) Progesterone (P4) and estrogen (E2) concentration change comparison among control and heat stress groups (E and F, respectively). ELISA-based calorimetric measurement of P4 and E2 in control and treatment groups of vitamin C. Data are represented as mean ± S.D. of at least three independent cultures with further at least three replicates for each culture. Bars with asterisk (*) symbols above them are significantly different (*p*-value < 0.05) from control (38 °C) group, while bars with hash (#) symbols are significantly different (*p*-value < 0.05) from heat stress (43 °C) group.

Vitamin C, as a cofactor of diverse biological functions in the body, also possesses anti-inflammatory and antioxidant functions. Therefore, we examined the cell viability and cell cycle progression of GCs after heat stress to assess how vitamin C helps GCs overcome the effects of heat stress. Similarly, in order to assess the state of ROS and apoptosis, we studied mitochondrial and cellular ROS and its final outcome on the cell apoptosis under vitamin C supplementation. Cultured GCs were exposed to heat stress treatment (43 °C) in vitro, while the control group remained at 38 °C. GCs were treated with varying concentrations of vitamin C dissolved in complete culture media. Cells in the control group maintained steady viability with a slight increase in viability through increasing doses of vitamin C. A significant increase (*p*-value < 0.05) in the viability of heat-stressed GCs was observed at 200 μM and 300 μM of vitamin C concentration. While there was no difference between 200 μM and 300 μM, 250 μM was selected as the effective ameliorating dose (Figure 1A) for subsequent experimentations.

**Figure 2:**
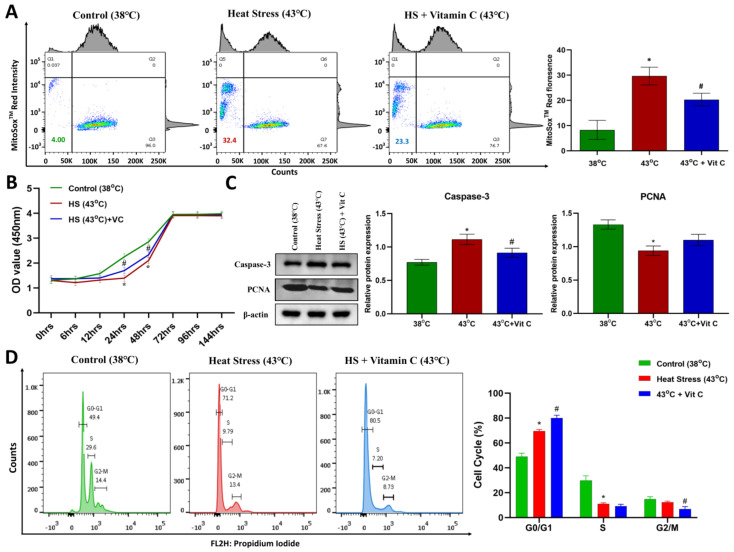
Physical parameters of bovine granulosa cells. Comparison of physical parameters of bovine granulosa cells (GCs) exposed to heat stress (43 °C for 2 h) and vitamin C (Vit C) treatment versus control (38 °C). (**A**) Representative red fluorescence of GCs stained with MitoSox^TM^ Red measured via flow cytometry. Relative fluorescence values are shown on the *y*-axis, and the treatments are indicated on the *x*-axis. (**B**) GCs viability assay-based cell growth assay for different time points with mean optical densities (OD) measured at 450 nm wavelength are plotted for control, heat stress and heat stress plus vitamin C groups. (**C**) For all treatment groups, the Caspase-3 and PCNA protein expression was detected by Western blotting in GCs. (**D**) Representative flow cytometry plots of cell cycle analysis in control group, heat stress and heat stress plus vitamin C groups, along with means comparison of cell cycle phases (G0/G1, S and G2/M) of GCs under all three groups. Data are represented as mean ± S.D. of at least three independent cultures with at least three further replicates for each culture. Bars with asterisk (*) symbols above them are significantly different (*p*-value < 0.05) from control (38 °C) group, while bars with hash (#) symbols are significantly different (*p*-value < 0.05) from heat stress (43 °C) group.

GCs with 250 μM vitamin C-containing culture media treatment were subjected to post-heat stress exposure recovery for 6 h at 38 °C, after which ROS and apoptosis in GCs were estimated. Compared to the control group, a significant (*p*-value < 0.05) increase in both mitochondrial and cytosolic ROS levels (Figure 1C and Figure 2A) was observed in the heat stress treatment group. Treatment of vitamin C has a significant (*p*-value < 0.05) negative effect on ROS production in acute heat-stressed GCs (Figure 1C and Figure 2A). Similarly, heat-stressed GCs had significantly higher (*p*-value < 0.05) apoptotic rates compared to the control group, as shown in representative flow cytometry plots of early apoptotic and late apoptotic events in the control, heat stress and heat stress plus vitamin C groups, respectively. The treatment of vitamin C significantly (*p*-value < 0.05) decreased the apoptosis rate in heat-stressed GCs (Figure 1B), which was also confirmed by the significantly (*p*-value < 0.05) decreased expression of Caspase-3 (Figure 2C). Progesterone and estrogen concentrations were significantly (*p*-value < 0.05) decreased in the culture media of the heat stress group, while a positive response was observed in the vitamin C treatment group (Figure 1D,E). Cell cycle analysis after six hours post-stress revealed that cells in the vitamin C group, when compared with the heat stress group, were significantly (*p*-value < 0.05) high in G0/G1 and significantly (*p*-value < 0.05) low in the G2-M stage of the cell cycle. Furthermore, after the conclusion of 2 h of treatment, we changed 80% of the respective culture media (medium with added vitamin C in heat stress plus vitamin C group) and performed a cell growth recovery assay for 144 h (Figure 2D). While cells in the control group maintained steady proliferation, the vitamin C treatment group with a high proliferation expression marker of PCNA protein, even at six hours post-stress (Figure 2C), had a clearly significant (*p*-value < 0.05) increased proliferation at 24 h and 48 h.

### 3.2. Metabolome Analysis

While ruminant animals can synthesize vitamin C by themselves, primates cannot, and they satisfy the body’s need for vitamin C through food intake. However, vitamin C is rapidly depleted in states of stress and disease, resulting in lower plasma ascorbic acid concentrations [10]. Similarly, while the ex vivo handling of cells makes them devoid of this systemic vitamin C support, together with the naturally depleted body states of vitamin C, it is important to study the ameliorating effects of vitamin C under heat stress. One of the main culprits behind the induction of cell apoptosis is the generation of cellular ROS, which itself is the product of abrupt metabolic reshuffle to external stressful stimuli. At the same time, the change in the cell cycle and the onset of cell senescence causes a transient halt in cell proliferation, which again originates from the pathways mediated by metabolic reshuffle. Therefore, we performed a metabolomics investigation to dissect and elaborate on these metabolic components and pathways. Untargeted metabolome analysis in triplicate was performed for both treatment groups involving vitamin C through LC-MS/MS. Metabolite tables of both negative and positive ion modes were each used for PCA analysis (Supplementary Figure S1), displaying a clear separation between the two treatment groups involving vitamin C. In all treatment groups, 43 and 38 metabolites, along with their regulation status, are determined in positive (Table 1) and negative ion mode (Table 2), respectively.

**Figure 3:**
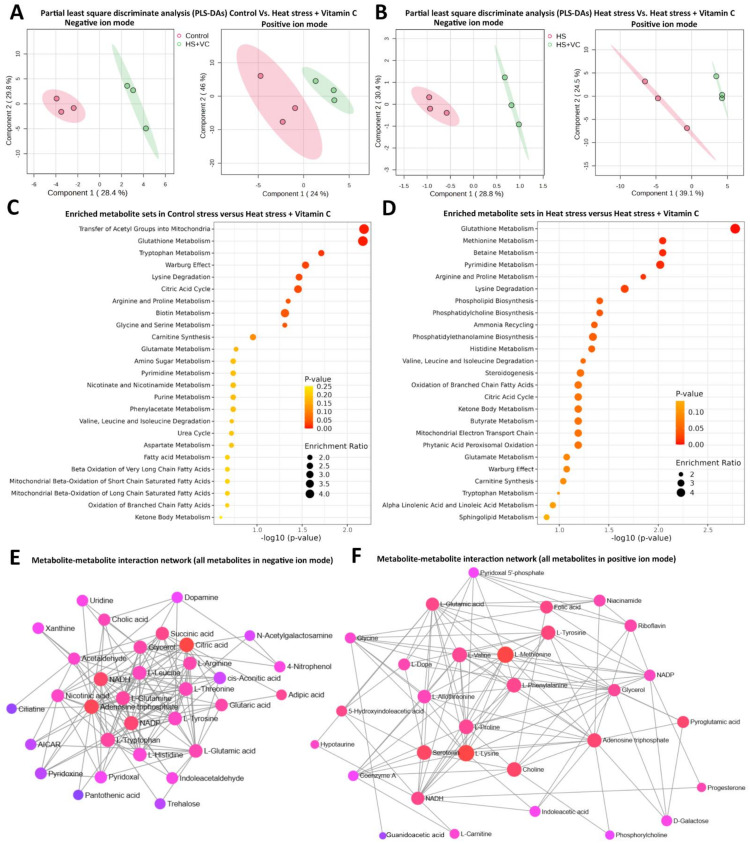
Results of metabolome analyses. (**A**,**B**) Partial least squares-discriminate analysis (PLS-DA) scores of metabolite sets in negative and positive ion modes for control versus heat stress plus vitamin C (**A**) and heat stress versus heat stress plus vitamin C (**B**). (**C**) Small molecule pathway database (SMPDB)-based enrichment analysis of metabolites with VIP score ≥ 1 (variable importance in the projection score) in both ion modes of control versus heat stress plus vitamin C. (**D**) SMPDB-based enrichment analysis of differentially expressed metabolites (DEMs) with VIP score ≥ 1 in both ion modes of heat stress versus heat stress plus vitamin C. (**E**) Metabolite–metabolite interaction network analysis of all DEMs in negative ion mode. (**F**) All DEMs in positive ion mode are subjected to metabolite–metabolite interaction network analysis with circular nodes (bright red being the most central ones and purple being the less important) and lines joining the nodes referred to as edges.

Partial least squares-discriminate analysis (PLS-DA) scores of metabolite sets in negative and positive ion modes for control versus heat stress plus vitamin C and heat stress versus heat stress plus vitamin C are presented in Figure 3A,B, respectively. An optimum distinction between both groups in each comparison is evident. A total of 17 DEMs were determined in each comparison both in positive and negative ion modes (and used in downstream analyses), as presented in Table 1 and Table 2, where the majority of them belong to organic compounds, amino acids and their derivatives and amines. Moreover, 15 DEMs appeared to be common in two or all three comparisons; however, common DEMs in both comparisons involving vitamin C include Indole-acetic acid, Aminoadipic acid, Urocanic acid, 16-Hydroxyhexadecanoic acid, Ciliatine, Succinic acid and Uridine. Significantly differential metabolites in both modes were identified (*p* < 0.05) and marked with an asterisk along with VIP scores. Furthermore, all DEMs in both comparisons involving vitamin C were subjected to KEGG-based metabolite–metabolite interaction network analysis (Figure 3E,F), where circular nodes are the metabolites with varying importance in the given network (bright red being the most central ones and purple being the less important). The statistical scores of each metabolite in the network (nodes) and the interacting lines (edges) are based on betweenness and centrality values in the network (given in Supplementary Table S1).

**Figure 4:**
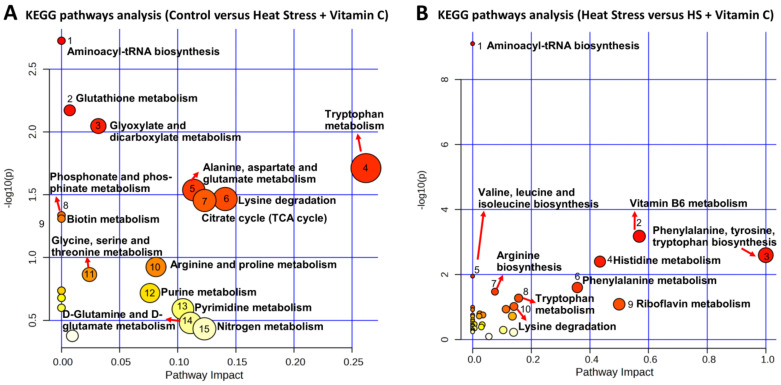
Kyoto Encyclopedia of Genes and Genomes (KEGG)-based metabolic pathway enrichment analysis using differential metabolites in both ion modes of control versus heat stress plus vitamin C (**A**) and heat stress versus heat stress plus vitamin C (**B**). Important pathways are labeled.

Furthermore, all DEMs in control versus heat stress plus vitamin C and heat stress versus heat stress plus vitamin C, irrespective of their ion modes, were subjected to SMPDB- (Figure 3C and Figure 4A) and KEGG (Figure 3D and Figure 4B)-based pathway enrichment analysis. The detailed information related to enriched metabolite sets in both comparisons is given in Supplementary Table S2 for SMPDB enrichment analysis and in Supplementary Table S3 for KEGG pathway analysis. Glutathione metabolism appeared to be the single largest enriched metabolic pathway not only in both comparisons but also in both types of pathway analyses, where Pyroglutamic acid (significantly differential metabolite) was enriched with an upregulation in control versus heat stress and increasing downregulation trend in both comparisons involving vitamin C. Similarly, Lysine degradation was another pathway common in both comparisons with Lysine being upregulated in control versus heat stress plus vitamin C and downregulated in heat stress versus heat stress plus vitamin C, while the L-2-Aminoadipic acid increasingly upregulated in both comparisons. The tryptophan metabolism pathway was found to be a highly enriched pathway in the control versus heat stress plus vitamin C with exclusively downregulated Tryptophan and upregulated Indole–acetic acid and Indole acetaldehyde. A highly enriched Warburg effect pathway shows the signature high rate of oxidative metabolism in heat stress. The citric acid cycle was the highly enriched pathway in control versus heat stress plus vitamin C comparison of both SMPDB and KEGG pathway enrichment analyses with upregulated Citric acid and Succinic acid. KEGG pathway analysis yielded three additional pathways related to B-complex vitamins, including vitamin B6 metabolism, Riboflavin metabolism and Biotin metabolism.

**Figure 5:**
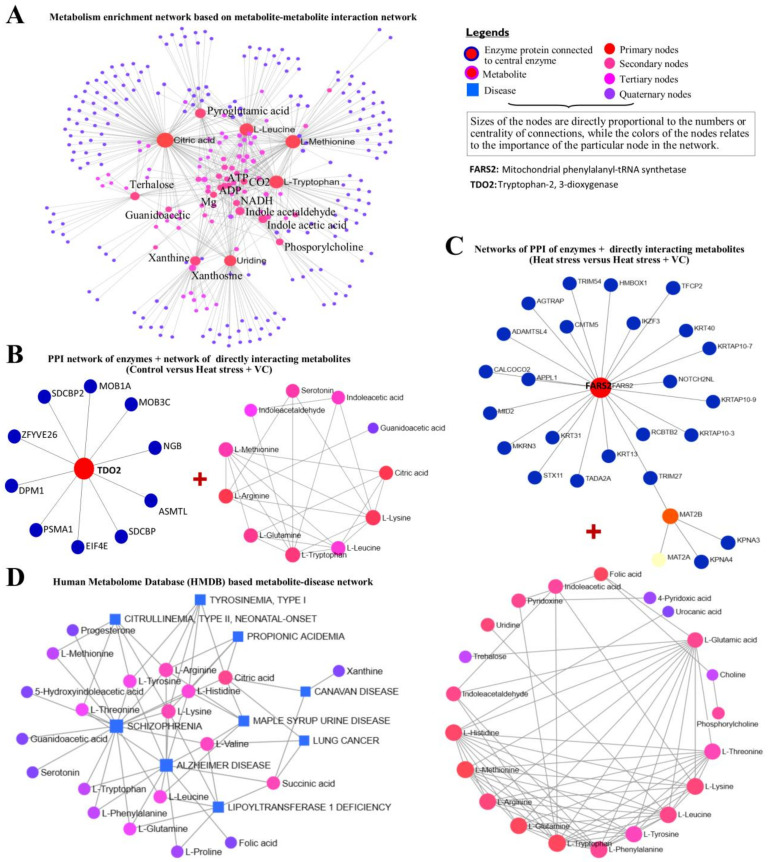
Kyoto Encyclopedia of Genes and Genomes (KEGG)-based central metabolites interaction network using differential metabolites from both control versus heat stress plus vitamin C and heat stress versus heat stress plus vitamin C (**A**). (**B**) Protein–protein interaction (PPI) network of enzyme proteins based on differential metabolites along with related metabolites interaction network in control versus heat stress plus vitamin C. (**C**) PPI network of enzyme proteins based on differential metabolites along with related metabolites interaction network in heat stress versus heat stress plus vitamin C. (**D**) Disease and metabolite network illustration based on the differential metabolites found in both comparisons involving vitamin C.

At the same time, the most important metabolites from both heat stress groups were separately subjected to KEGG-based qualitative network analysis (Figure 5A), where Citric acid, L-Methionine, L-Leucine, Pyroglutamic acid and L-Tryptophan were the most important differential metabolites. Additionally, PPI networks of enriched enzymes (details are given in Supplementary Table S1) and corresponding metabolite interaction networks in both comparisons involving vitamin C were generated for control versus heat stress plus vitamin C (Figure 5B) and heat stress versus heat stress plus vitamin C (Figure 5C). Tryptophan 2,3-dioxygenase (TDO2) was the central network node enriched in control versus heat stress plus vitamin C with L-arginine, L-lysine, Citric acid and L-glutamine as the important interacting metabolites. At the same time, mitochondrial phenylalanyl-tRNA synthetase (FARS2) was the central enzyme protein node along with L-methionine, L-tryptophan, L-histidine, L-glutamine, L-arginine, L-leucine and L-lysine as the main interacting metabolites in heat stress versus heat stress plus vitamin C group. Furthermore, KEGG-based joint disease and metabolite network analysis was performed using input of DEMs enriched in metabolic pathways (Figure 5D), while the details of this interaction network are given in Supplementary Table S2. Wherever Alzheimer’s disease and Schizophrenia were the two major diseases, Citric acid, Succinic acid, L-lysine, L-threonine, L-arginine, L-leucine and L-histidine were the major interacting metabolites.

## 4. Discussion

Ovarian GCs have an integral role in reproduction through their steroidogenic and oocyte development roles [29,30]. Additionally, during the events of ex vivo handling and culturing conditions, investigation of vitamin C ameliorative properties and underlying mechanisms using GCs present insightful simulation in the context of reproductive biotechnologies. Therefore, it was worth undertaking to investigate the biochemical and metabolome changes in in vitro acute heat stressed GCs supplemented with vitamin C. 

An increase in the colorimetric OD values at 48 h post-stress is indicative of transient proliferation senescence in GCs and implies that cell viability and proliferation can be improved through antioxidant support [31,32]. This positive effect of vitamin C supplementation on cell viability is in accordance with the previous studies performed in vitro [33,34]. In GCs of sows, PCNA is reported as one of the key proteins of cell proliferation potential under natural conditions and mainly implicates in the S phase of the cell cycle during stress and apoptosis [35,36,37]; the addition of vitamin C to heat-stressed GCs considerably increased the proportion of this protein in our study. Our results revealed that the heat stress caused transient quiescence in the form of cell cycle arrest at the G0/G1 phase, which was even higher in the vitamin C group. Many reasons can be argued for this unusual phenomenon. This resting quiescence-type phenotype can be described as an interphase of transient cell senescence of GCs [31,32]. The second reason pertains to the extreme stress and possible nuclear deformation, which also coincides with S and G2/M phase phenotype (though statistically significant but it cannot be construed as absolutely true) observed in our study can be best explained by the fact that extreme cellular stress can impact the attributes of nuclei during the G2/M phase [37,38], thus affecting the PI staining. For instance, when cells are in G2, they contain about twice the DNA content as in G1, which would normally mean they take up more dye and fluoresce more brightly. However, if the nuclei are deformed due to heat stress [38], this staining might not precisely depict the DNA content of the cell. Therefore, it is recommended to carry out comprehensive and multi-time points measurements of cell cycle phases under heat stress and vitamin C conditions in order to establish cell phenotype. Acute heat stress caused intracellular ROS accumulation and apoptosis of GCs in accordance with the prior studies [39,40], and these effects were significantly ameliorated with the supplementation of vitamin C. Heat stress caused cellular ROS to trigger oxidative stress [41] and the apoptosis of cells [42,43]. Studies report the implication of the Nrf2 (nuclear factor (erythroid-derived 2)-like 2) pathway in upregulating the cellular antioxidant responses for the protection of cell apoptosis [40,44,45]. Under cell stress conditions, the Nrf2 pathway also regulates pathway functions related to cell metabolism in conjunction with PI3K/AKT/mTOR [46,47,48] and AMPK signaling pathways [46,49]. Therefore, mechanistic studies of these pathways under heat stress and vitamin C supplementation seem imperative and would unlock the mechanisms of metabolic regulation and its ultimate relationship with cell fate. Heat stress decreased the production of progesterone and estrogen hormones, as demonstrated previously [39,44]. Nevertheless, the vitamin C supplementation to the cells significantly restored these hormones’ concentration. Results of metabolomics confirmed the rise of progesterone in heat-stressed GCs supplemented with vitamin C. The role of GCs is critical in driving the formation of ovarian follicles and building the cumulus–oocyte complex surrounding the oocyte, and there is a specialized crosstalk between GCs and oocyte which is important both for the natural fertility and assisted reproduction technology (ART) success [50]. In one study performed in the Sertoli cell line culture, VC is shown to decrease oxidative stress and, thereby, protect cells from the adverse effects of HS [34]. Amelioration of heat stress associated with endoplasmic reticulum stress and unfolded protein response signaling pathway can be the other cause of the improvement in the level of progesterone concentration in GCs [44]. There are no definite studies on this nexus of steroidogenesis in heat-stressed GCs; however, this may be due to the transcription level changes because the cholesterol, being the master precursor of progesterone, also experiences a decrease with acute heat stress [19,51,52]. Exhaustive mechanistic studies on the role of the unfolded protein response signaling pathway and its relationship with steroidogenesis will certainly help to understand these mechanisms and provide further intervention opportunities.

**Figure 6:**
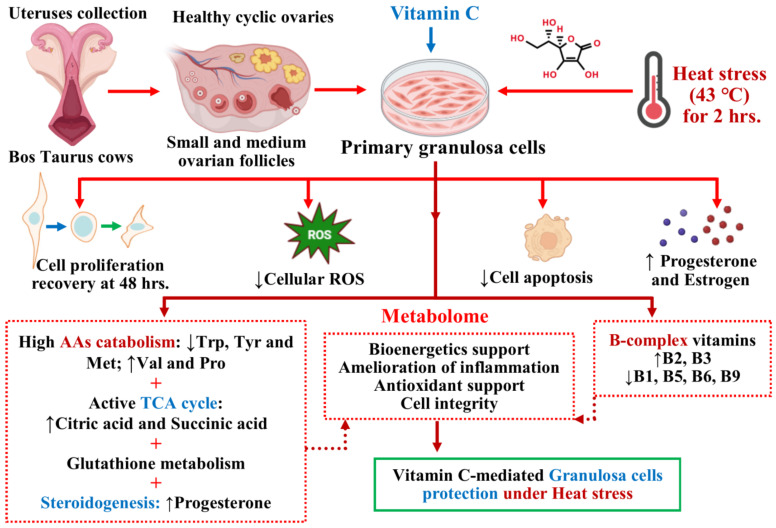
Summary of results and major metabolic changes attributed to vitamin C-mediated protection of the granulosa under in vitro heat stress.

This characterization of the metabolic profile of heat-stressed GCs in the presence of exogenous vitamin C presents a valuable simulation of the ovarian follicular microenvironment under heat-stress conditions. Some of the most abundant molecules were involved in amino acids, carbohydrates and lipid metabolism. Among amino acids, Tryptophan was exclusively downregulated in all comparisons, followed by L-tyrosine, Methionine and L-leucine. L-leucine and Glutamine levels particularly tend to go down in the presence of vitamin C. With the supplementation of vitamin C, heat-stressed GCs show a gradual increase in the levels of Valine and Proline. Surprisingly, L-leucine, Glutamine, Threonine, Lysine and Arginine had the same pattern of regulation in the presence of vitamin C treatment. Tryptophan is shown to decrease in response to acute heat stress [53]. An increase in the level of L-leucine has been shown to be the marker of heat stress [54], which certainly did increase heat stress; however, supplementation with vitamin C significantly dragged down its levels in our study. Amino acids should follow anabolic or catabolic pathways, and heat stress triggers catabolism to meet high energy demands [55,56]. Therefore, the changes in free amino acid levels may be assumed to be the result of catabolic processes [53,57]. Catabolic pathways involve deamination or deamidation followed by either reanimation to non-essential amino acids (Proline was upregulated in response to vitamin C) or direct channeling into the TCA cycle, where most likely they become oxidized or channeled towards gluconeogenesis via pyruvate carboxylase. Tryptophan metabolism was the significantly enriched metabolic pathway in response to vitamin C with low Tryptophan and high Indole–acetic acid, Indole acetaldehyde and Mycophenolic acid. On the other hand, levels of Niacin and Serotonin increased in the presence of decreased Tryptophan. The enrichment of mitochondrial phenylalanyl-tRNA synthetase (FARS2) and 2,3-dioxygenase (TDO2) enzymes in response to vitamin C supplementation is the indication of simultaneous catabolic and regulatory molecular dogma implicated in vitamin C-mediated protection of heat-stressed GCs. The interaction of 2,3-dioxygenase and Tryptophan metabolism has an established anti-inflammatory role in cells through suppressing cytokine signaling and cell protection roles via Serotonin and Niacin [58,59]. There was evidence of preferential amino acid catabolism being promoted by vitamin C under HS; however, it is worth mentioning that the catabolism of some amino acids also plays regulatory roles through cell signaling and modulating the inflammation–metabolism nexus [60,61]. Methionine metabolism and Lysine degradation were the following important metabolic pathways, not to mention that Methionine and Lysine were the two most important metabolites enriched in metabolite–metabolite network. Glutathione metabolism was the single largest enriched metabolic pathway in both comparisons involving vitamin C, whereas Pyroglutamic acid increased in heat stress alone but experienced a decrease when accompanied by vitamin C. This phenomenon indicates an interrelationship between vitamin C and Glutathione and may be the main cause of the protection of GCs from adverse effects of heat stress, as both of them are shown to exert an additive antioxidant effect [62]. Most importantly, Citric acid (central to the TCA cycle) was significantly upregulated in vitamin C-supplemented heat-stressed GCs, related to the catabolism of fatty acids, carbohydrates and amino acids, which presents a classic example of catabolic activities’ restoration compared to the non-supplemental GCs group. Citric acid is central to diverse cellular energy support [63] and helpful in relieving oxidative stress and [64] heat stress in tissues [65]. Succinic acid as the intermediate of Citric acid synthesis was also significantly increased, readily showing sugar availability for the bioenergetic support of heat-stressed GCs supplemented with vitamin C. Evidence from our study suggests less likeliness of conversion for fatty acid synthesis in heat stress with the exception of gamma-Linolenic acid, which increased in response to vitamin C supplementation. Gamma-Linolenic acid supplementation is shown to protect cells from heat stress damage and rescuing cellular antioxidant support [66]. Additionally, there is less evidence of β-oxidation in the form of decreased L-carnitine in response to vitamin C under heat stress [67]. Alzheimer’s disease and Schizophrenia were the two major diseases found in joint metabolome networks of differential metabolite, where aside from fatty acid metabolism, abnormality in amino acid and glutamate metabolism is related to the regulation of metabolites in heat stress and energy-buffering systems in GCs and mammals [16,56,68]. Vitamin C ameliorates the negative effects of heat stress associated with the inflammation–metabolism nexus together with the correction of antioxidant status. Therefore, it can be construed from our results that this might be of relevance as these diseases are also governed by the inflammation-and-metabolism nexus together with the disturbed redox status. Vitamin B2 obviously increased, while interestingly, vitamin B5 and vitamin B6 decreased in response to vitamin C supplementation. Similarly, vitamin B3 increased (Tryptophan remained decreased), and vitamin B9 and B1 decreased in response to vitamin C supplementation to heat-stressed GCs. Members of the vitamin B complex are integral for the cellular processes in a variety of precursor, cofactor, coenzyme and substrate roles [69]; this trend of B-complex vitamin regulation clearly shows a shift from an anabolic to catabolic milieu [70,71]. The increase in hydrophilic vitamins B2 and B3 further confirms the preferential catabolism of amino acids for cellular energetic support [70,72]. Nevertheless, further restriction and rescue experiments involving these implicated amino acids and B vitamins in cellular and animal models under heat stress will help to understand and optimize relevant support remedies under heat stress. A summary of the results and major metabolic changes attributed to the vitamin C-mediated protection of the granulosa under heat stress is illustrated in Figure 6.

## 5. Conclusions

In conclusion, this study comprehended the broad metabolic milieu of heat stressed bovine GCs with or without supplementation of the vitamin C. Vitamin C supplementation proved to be a versatile antioxidant support remedy in the event of heat stress. Cellular energetic metabolism is severely strained in heat stress; besides relieving oxidative stress, vitamin C supplementation modulates cellular catabolism in a way that confers maximum protection and less loss of function in the GCs. Evidence of amino acid catabolism is the most prominent, and the significance of various amino acids like Tryptophan, Methionine, Leucine and Tyrosine is obvious in the context of this study. The current study presents metabolic intervention faucets in conjunction with the importance of antioxidant support in the event of heat stress. One can also infer the importance of progesterone, essential amino acids, vitamin B complex, supplemental fatty acids and antioxidant support to combat adverse effects of heat stress, enhance fertility rates and augment the success of ART. Nevertheless, this study also underscores the importance of further mechanistic studies at the metabolic nexus to precisely optimize the exogenous antioxidant support at cellular and organismal levels in the events of stressful stimuli.

## Figures and Tables

**Table 1:** List of metabolites determined from the positive ion mode of LC-MS/MS analysis by using all treatment groups. Regulation status and partial least square discriminate analysis (PLS-DA)-based variable important in projection (VIP) values along with *t*-test-based significance of every metabolite in each comparison is detailed.

Metabolites (Positive Ion-Mode)	C vs. HS		C vs. HS + VC		HS vs. HS + VC	
	Log2(FC) VIP		Log2(FC) VIP		Log2(FC) VIP	
(-)-Riboflavin	−0.404	1.489	0.007	0.287	0.421	1.145
1-Amino-1-cyclopentanecarboxylic acid	0.015	0.850	0.226	1.713	0.210	0.860
1-Methylhistamine	−0.054	0.970	0.090	0.624	0.148	1.554
3,4-Dihydrocoumarin	0.037	0.964 *	0.034	0.479	0.006	0.274
3-Formylindole	0.031	0.754	0.166	0.853	0.150	0.588
4-Acetamidoantipyrine	−0.423	1.454	0.101	0.724	0.517	1.559 *
4-Hydroxybenzoylcholine	0.208	0.485	0.004	0.499	−0.188	1.406
5-Hydroxyindole-3-acetic acid	0.076	0.688	0.093	0.586	0.041	0.145
Aspartame	−0.038	1.093 *	−0.138	0.570	−0.087	0.568
beta-Guanidinopropionic acid	−0.199	1.361 *	−0.301	1.395	−0.099	0.445
Choline	1.113	1.588 *	0.201	0.584	−0.895	1.528 *
D-(+)-Galactosamine	0.323	0.023 *	−0.099	0.235	−0.421	1.666
D-(+)-Glucosamine	0.155	0.459	0.095	0.707	−0.071	0.062
DOPA	0.137	0.579	−0.344	1.305	−0.463	0.789
Folic Acid	−0.429	1.295	0.077	0.503	0.490	1.235
Galactose	0.177	0.497 *	0.032	0.663	−0.148	0.663
Gentiobiose	0.632	0.281	−0.393	1.974	−0.999	1.251
Glycocyamine	−0.304	1.501 *	−0.340	1.493	−0.033	0.190
Hexylamine	0.030	0.988 *	0.015	0.426	−0.003	0.232
Hypotaurine	−0.494	0.873	0.362	0.632	0.819	0.854
Indoleacetic acid	−0.092	0.955	0.263	1.181	0.355	1.422
L-2-Aminoadipic acid	−0.303	1.230	0.319	1.164	0.622	1.763 *
L-5-Oxoproline	0.107	0.760	−0.551	2.141 *	−0.642	1.769 *
L-allo-Threonine	0.164	0.728 *	0.182	1.703	0.027	0.328
L-Carnitine	−0.358	1.389	−0.497	1.310	−0.116	0.498
Levetiracetam	−0.386	1.136	0.136	0.610	0.521	1.022
Lysine	0.344	0.067	0.098	1.456	−0.231	0.636
Methionine	0.097	0.632	−0.353	1.692	−0.431	1.074
Mycophenolic acid	0.167	0.615	0.146	1.164 *	−0.016	1.190
Niacinamide	−0.008	0.814	0.278	0.981	0.283	0.418
Phenanthridine	0.249	0.313	0.023	0.366	−0.223	1.143
Phenylalanine	0.055	0.939 *	0.027	0.489	−0.017	0.099
Phosphocholine	−0.890	0.832	0.350	1.043	1.174	1.282
Progesterone	−1.281	1.747	−0.493	0.281	0.769	1.471
Proline	−0.218	1.323 *	0.058	0.589	0.278	1.401
Pyrimidinol	0.261	0.140	0.046	1.401	−0.196	0.605
Salsolinol	−0.066	1.101 *	−0.183	0.524	−0.106	0.594
Serotonin	0.169	0.515	0.182	1.108	0.014	0.072
Thiamine	−0.127	1.134	−0.179	0.525	−0.035	0.071
Tyrosine	0.150	0.567	−0.065	0.127	−0.204	0.722
Urocanic acid	−0.588	1.351	0.383	1.011	0.957	1.569 *
Valine	−0.190	1.086	0.042	0.281	0.244	0.616

(*) asterisks show the metabolite with Student’s *t*-test-based significance of *p* < 0.05 in respective comparison. DOPA: dopamine. VIP stands for variable important in projection. VIP ≥ 1 are referred to as differentially expressed metabolites (DEMs). Control versus heat stress (C vs. HS) control versus heat stress plus vitamin C (C vs. HS + Vit C), and heat stress versus heat stress plus vitamin C (HS vs. HS + Vit C).

**Table 2:** List of metabolites determined from the negative ion mode of LC-MS/MS analysis from using treatment groups. Regulation status and partial least square discriminate analysis (PLS-DA)-based variable important in projection (VIP) values along with *t*-test-based significance of every metabolite in each comparison is detailed.

Metabolites (Negative Ion-Mode)	C vs. HS		C vs. HS + VC		HS vs. HS + VC	
	Log2(FC)	VIP	Log2(FC)	VIP	Log2(FC)	VIP
(-)-Citramalic acid	−0.127	0.712	0.228	1.102	0.348	1.561
16-Hydroxyhexadecanoic acid	−0.367	1.523 *	−0.236	1.848 *	0.123	1.019 *
2-Hydroxyisobutyric acid	−0.048	0.289	0.488	1.439	0.528	1.902
2-Hydroxyisocaproic acid	−0.058	0.316	−0.219	1.243	−0.168	0.927
3-Hydroxy-3-methylglutaric acid	0.197	1.097 *	0.148	1.443	−0.058	0.495
3-Indoxyl sulfate	−0.139	0.866 *	−0.195	1.641 *	−0.064	0.695
4-Nitrophenol	0.160	0.845	0.186	1.332	0.016	0.096
AICAR	−0.287	1.169	0.057	0.432	0.333	1.634 *
Cholic acid	−0.585	1.558	−0.216	0.561	0.362	1.310
Ciliatine	−1.324	2.741 *	−1.097	1.672 *	0.217	0.953
cis-Aconitic acid	−0.255	0.875	−0.010	0.009	0.237	1.115
Citric acid	0.359	1.333	0.505	1.592 *	0.139	0.756
D-(−)-Quinic acid	−0.198	0.944	−0.177	0.894	0.010	0.039
D-(+)-Pantothenic acid	−0.225	0.948	−0.104	0.685	0.113	0.596
D-(+)-Trehalose	−0.341	0.938	−0.256	0.607	0.072	0.055
DL-3-(4-Hydroxyphenyl)lactic acid	−0.383	1.293	0.052	0.020	0.428	1.412
gamma-Linolenic acid	−0.107	0.247	0.348	0.921	0.442	1.592
Glutamine	0.159	0.903	0.147	1.226	−0.022	0.200
Glutaric acid	−0.102	0.707	−0.021	0.640	0.072	0.700
Indole-3-acetaldehyde	0.357	1.338	0.387	1.160	0.021	0.023
Indole-3-carboxyaldehyde	0.095	0.301	0.702	1.295	0.598	1.781
L-(−)-Mandelic acid	−0.322	1.394 *	−0.042	1.788 *	0.041	0.380
L-(+)-Arginine	0.261	0.984	0.221	0.977	−0.050	0.257
L-Histidine	0.111	0.511	0.154	0.646	0.035	0.113
L-Iditol	−0.104	0.722	−0.104	1.374	−0.008	0.090
L-Leucine	0.298	1.272 *	0.075	0.584	−0.231	1.349 *
L-Norvaline	0.114	0.577	−0.114	0.638	−0.238	1.272
L-Tryptophan	−0.042	0.282	−0.270	1.763 *	−0.238	1.366 *
L-Tyrosine	0.230	1.005	−0.003	0.024	−0.240	1.241
Nicotinic acid	0.100	0.532	0.044	0.617	−0.065	0.453
N-Tigloylglycine	−0.091	0.452	−0.176	1.332	−0.092	0.375
Pyridoxal	0.168	0.704	0.097	0.665	−0.081	0.382
Pyridoxine	0.331	1.094	−0.037	0.266	−0.380	1.407
Sebacic acid	0.179	0.974	0.120	1.212	−0.069	0.577
Succinic acid	−0.259	1.162 *	0.331	0.993	0.582	1.985
Threonine	0.169	0.878	0.083	0.668	−0.095	0.640
Uridine	−0.913	2.182 *	−0.302	0.756	0.602	2.153 *
Xanthine	0.324	1.231	−0.198	0.891	−0.450	1.819

(*) asterisks show the metabolite with Student’s *t*-test-based significance of *p* < 0.05 in respective comparison. AICAR: 5-Aminoimidazole-4-carboxamide-1-beta-D-ribofuranosyl 5′-monophosphate. VIP stands for variable important in projection. VIP ≥ 1 are referred to as differentially expressed metabolites (DEMs). Control versus heat stress (C vs. HS), control versus heat stress plus vitamin C (C vs. HS + Vit C) and heat stress versus heat stress plus vitamin C (HS vs. HS + Vit C).

## Data Availability

Untargeted LCMS-MS spectra data of this study have been deposited in the National Genomics Data Center-China National Center of Bioinformation (NGDC-CNCB) with the accession number PRJCA025452.References.

## Supplementary Materials

The following supporting information can be downloaded at: https://www.mdpi.com/article/10.3390/antiox13060653/s1. Supplementary Figure S1. Metabolites in both positive and negative ion mode found in each comparison involving vitamin C were separately subjected to principal component analysis (PCA) analysis, which displays a clear separation between the two treatment groups involving vitamin C. Supplementary Table S1. Summary of statistics of all interaction network analyses performed and mentioned in main figures of the manuscript, including Figure 3E,F and Figure 5A–D. Supplementary Table S2. Small molecule pathway database (SMPDB)-based metabolite enrichment analysis in control versus heat stress plus vitamin C (1) and heat stress versus heat stress plus vitamin C (2). Supplementary Table S3: Summary statistics of Kyoto Encyclopedia of Genes and Genomes (KEGG)-based pathway analyses of differentially expressed metabolites in both comparisons involving vitamin C.
