# Supplementary material for: Vitamin C Alleviates the Negative Effects of Heat Stress on Reproductive Processes by Regulating Amino Acid Metabolism in Granulosa Cells
Source: Antioxidants (Basel). 2024 May 27;13(6):653. doi: 10.3390/antiox13060653 (PMC11201207; doi:10.3390/antiox13060653)
Supplement: Supplementary file 1 [file antioxidants-13-00653-s001.zip › Supplementary figure S1.pdf]

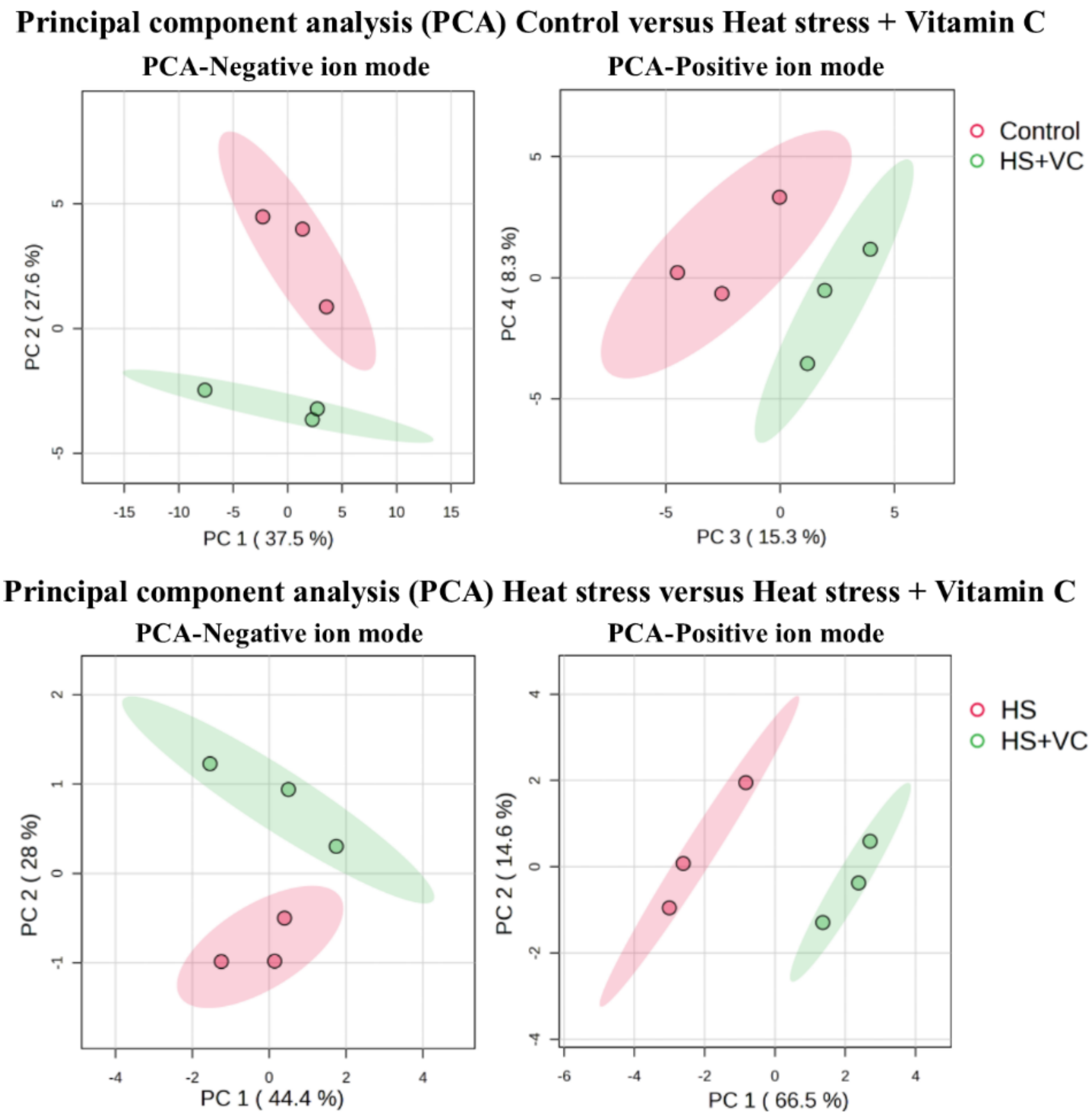

Supplementary figure 1. Metabolites in both positive and negative ion mode found in each comparison involving vitamin C were separately subjected to principal component analysis (PCA) analysis, which displays a clear separation between the two treatment groups involving vitamin C.
